# Cervical bacterial colonization in women with preterm premature rupture of membrane and pregnancy outcomes: A cohort study

**Published:** 2018-05

**Authors:** Nafiseh Saghafi, Leila Pourali, Kiarash Ghazvini, Asieh Maleki, Mahdis Ghavidel, Mohsen Karbalaeizadeh Babaki

**Affiliations:** 1 *Department of Obstetrics and Gynecology, Faculty of Medicine, Mashhad University of Medical Sciences, Mashhad, Iran. *; 2 *Department of Microbiology, Faculty of Medicine, Mashhad University of Medical Sciences, Mashhad, Iran. *; 3 *Department of Medical Bacteriology and Virology, Faculty of Medicine, Mashhad University of Medical Sciences, Mashhad, Iran.*

**Keywords:** Bacterial colonization, Genital tract, Preterm premature rupture of membrane

## Abstract

**Background:**

One of the most important etiologies in pretermpremature rupture of membranes (PPROM) is cervical bacterial colonization.

**Objective:**

This study evaluated cervical bacterial colonization in women with PPROM and the pregnancy outcomes.

**Materials and Methods:**

In this cohort study, 200 pregnant women with PPROM at 27-37 wk of gestation who were admitted in an academic hospital of Mashhad University of Medical Sciences from March 2015 to July 2016 were studied. samples were obtained from endocervical canal for detection of routine bacteria and Gram staining. Also, we obtained one blood culture from neonates. Maternal endocervical culture, chorioamnionitis, neonatal intensive care unit admission, neonatal positive blood culture, neonatal sepsis, and mortality were documented.

**Results:**

Most common isolated microorganism of endocervical culture were Escherichia coli (24.2%), Coagulase negative Staphylococci (27.2%), Enterococcus and candida each one (11.7%). The prevalence of GBS was only 2.2%. Simultaneous positive blood cultures were seen in 3% of neonates. Among them, Gram-negative bacilli accounted for (66.6%), while Gram-positive cocci and candida made up only (16.7%). Endocervical colonization was associated with a higher admission rate (p=0.004), but there was no significant correlation between endocervical colonization and chorioamnionitis, positive blood culture and neonatal mortality rate.

**Conclusion:**

With regard to low GBS colonization rate, appropriate antibiotic regimens should be considered in PPROM cases according to the most prevalent micro organisms of endocervical bacterial colonization. Maybe cervical bacterial colonization had some effects on neonatal outcomes. There was no significant association between endocervical bacterial colonization and chorioamnionitis, positive neonatal blood culture and neonatal mortality.

## Introduction

Premature rupture of membrane (PROM) is defined as membrane rupture before the beginning of labor contractions and if it happens before 37 wk of gestation, it is called preterm premature rupture of membrane (PPROM) which is one of the main cause of preterm labor and neonatal death in about 2.3% of newborns (1, 2). Although neonatal mortality has declined in current century, preterm labor is still one of the main causes of prenatal mortality and neurologic morbidities among these neonates (3, 4). There is some evidence which showed the significant role of intrauterine infection in PROM cases (5, 6). Actually, in one-third of women with PROM; amniotic fluid cultures were positive. The imbalance vaginal bacterial colonization in pregnancy makes these women susceptible to colonization of pathogenic organisms (7).

PROM can cause maternal complications like chorioamnionitis, sepsis, placenta abruption, and endometritis. Prematurity as the most important neonatal complication increased the neonatal morbidity and mortality. In some studies, there were relationships between PPROM and intraventricular hemorrhage, periventricular leukomalacia and cerebral palsy (8-12). With regard to infectious causes in PPROM, antibiotics therapy is the main treatment for decreased maternal and neonatal complications like chorioamnionitis and neonatal sepsis (1, 13-15).

Group B streptococcus (GBS) is the main pathogen which its colonization has been observed in lots of western maternal genital tract in PPROM cases, also this is the main cause of early neonatal sepsis in these countries (16-18). So, American and Canadian guidelines have recommended the prophylactic administration of Ampicillin in these cases (16). But some studies in other countries have demonstrated complete different results which showed the presence of other microorganisms as the main pathogens of PPROM (16, 19-23). Although there are some studies which evaluated the distribution of endocervical bacterial colonization in Iranian women with PROM, the results were limited by small sample size or methodological shortage (24). 

With regard to the importance of maternal genital tract colonization as an etiologic factor in PPROM, appropriate antibiotic therapy has a cardinal role in prevention and treatment of maternal and neonatal complications. The aim of this study was the evaluation of bacterial colonization in genital tract of pregnant women with PPROM and its relationship with maternal and neonatal complications.

## Materials and methods

In this cohort study, 200 pregnant women with PPROM between 27-37 wk of gestation were studied in an academic Hospital in Mashhad University of Medical Sciences, Mashhad, Iran from March 2015 to July 2016. 

Diagnosis of membrane rupture was accomplished by speculum examination to see amniotic fluid leakage from cervical os and nitrazin test was done to confirm the diagnosis if needed. Inclusion criteria were PPROM, gestational age between 27-37 wk, no symptom or sign of chorioamnionitis at admission, and no antibiotics use before culturing. Exclusion criteriawas: patients’ refusal for being in the study. After exposing the cervix by sterile speculum, two samples were obtained with soft cotton swabs from endocervical canal; first swab was placed in Trypticase Soy Broth media and send immediately to the laboratory for detection of routine bacteria and Gram staining. This swab was cultured on sheep blood agar and Eosin methylene blue and evaluated after 24 incubation period. 

The second swab was placed in Lim Broth media and transported to laboratory and was incubated into a Todd Hewitt broth as selective enrichment broth with selective antibiotics in 5% CO_2_ at 35^o^C for 24-48 hr. After incubation, the enrichment broth is subcultured to blood agar plates and GBS like colonies are identified by the CAMP test and hydrolyzing ability for hippurate (25).

Also, we obtained one blood culture from neonates. After endocervical sampling for cases between 27-24 wk, we administered oral Azithromycin 1 gr (single dose) + intravenous Ampicillin 2 gr every 6 hr until 48 hr and the oral antibiotics (amoxicillin 500 mg every 8 hr) were administered up to 5 days if labor was not happened and two doses of 12 mg intramuscular betamethasone was also administered (12 mg every 24 hr for 2 doses). For cases between 34-36 wk of gestation, Ampicillin was administered as mentioned before and if labor didn’t start, pregnancy termination was planned after patient’s stabilization. Chorioamnionitis was defined as: temperature more than 38^o^C and presence of at least two below criteria:

1) Maternal tachycardia more than 100/min, 2) fetal tachycardia more than 160/min, 3) uterine tenderness, 4) malodor uterine discharge, 5) Maternal leukocytosis (WBC White blood cells >15000), and pregnancy termination was planned in these cases. Maternal chorioamnionitis, neonatal intensive care unit (NICU) admission, neonatal positive blood culture, neonatal sepsis (at first 3 days after delivery) and mortality were documented. With regard of the most frequent microorganism which growth in endocervical culture of PROM cases according to the study which performed in this academic hospital (9), Staphylococci epidermis was the most frequent microorganism (42%), so the sample size of 200 cases were calculated with regard of β=0.2 and α=0.05.

**Ethical consideration**

This study was approved by the Ethics Committee of Mashhad University of Medical Sciences, Mashhad, Iran (Ethical No. 5301588) and an informed written consent was taken from all the participants.

**Statistical analysis**

Data were analyzed by SPSS (statistical package for the social sciences, version 16.0, SPSS Inc, Chicago, Illinois, USA). With regard of the sample size in this study, parametric tests were used, so data analyzing was performed by Chi-square and Fisher Exact tests. p≤0.05 was considered statistically significant.

## Results

In this study, the age of participants was 15-42 yr with mean age of 27.6±2.3 yr. The mean gestational age was 31.5±2 wk. From 200 women; 101 women were primigravid (50.5%), 99 women were multi gravid (49.5%). The history of PROM in previous pregnancy was reported in 43.3% of cases. 21% of cases had comorbidities which diabetes (gestational and overt) was the most frequent.

**Figure 1:**
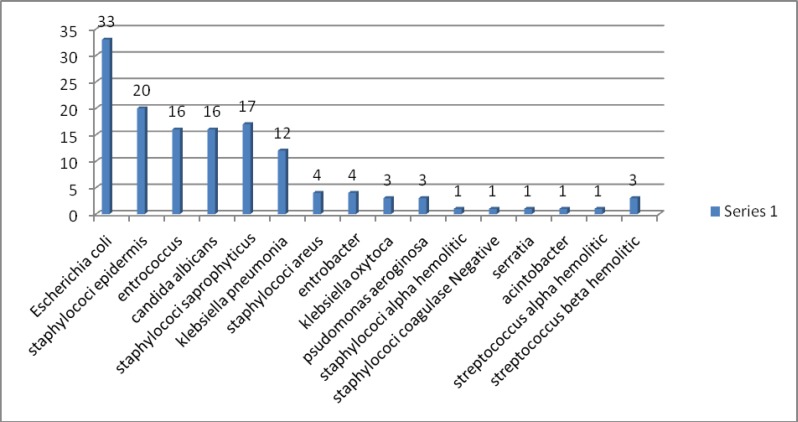
Microorganisms of endocervical culture of PPROM women

Evaluation of endocervical culture showed that 64 cases (32%) were culture negative and 136 cases (68%) were culture positive; which in 62 cases (31%) they were gram negative and in 58 cases (29%) they were gram positive microorganisms .in 16 cases (8%) fungal species were found. the endocervical cultures showed the most frequent pathogen was Escherichia coli (E. coli) 33 cases (24.2%) and after that Staphylococci epidermis (20 cases =14.7%); Staphylococci saprophyticus (17 cases =12.5%); Enterococcus saprophyticus and Candida species (16 cases in each group=11.7%) were the main pathogens. Just 3 cases (2.2%) of GBS were detected (Figure 1). 

There were 12 cases (12%) of maternal chorioamnionitis during expectant management, but Chi-square test didn’t show any significant relation between maternal chorioamnionitis and endocervical culture (Relative Risk (RR)=1.41, 95%CI: 0.39-5.3, p=0.59). From 12 chorioamnionitis cases; endocervical culture was negative in 3 cases (25%) and was positive in 9 cases (75%). Culture results showed the growth of Klebsiella pneumonia in 3 cases (24%), E. coli in 2 cases (17%), Enterococcus, Pseudomonas and Staphylococci epidermis and saprophyticus each in 1 case (8%). There was significant relation between chorioamnionitis and duration of PPROM (p<0.001) (Table I). 

In this study, we also evaluated the antibiogram for cultured microorganisms which showed that 54.4% of them were sensitive to at least one antibiotic group. The most sensitivity was related to penicillin (12.5%) and cefotaxime (11.7%). Just 2 microorganisms were sensitive to both penicillin and macrolide. About 50.8% of cultured microorganisms were resistant to at least one group of routine antibiotics which administered in PPROM cases (penicillin, cephalosporin, macrolide, and cefotaxime) which the most resistant (15.4%) was related to both penicillin and macrolide (Table II).

In this study, mean neonatal weight was 1500±200 gr. 95 neonates (47.5%) were admitted at NICU and 105 cases (52.5%) didn’t need NICU admission. NICU admission was significantly higher in women with positive endocervical culture (p=0.004) (Table III). Also, there was a significant relation between NICU admission and duration of membrane rupture till sampling (p<0.001). In this study all 200 women delivered and neonatal outcome was consisted of 180 live neonates (90%) and 20 neonatal death (10%). The causes of neonatal death were: 

1) Multiple congenital anomaly in 6 cases (30%); 

2) Respiratory distress syndrome in 5 cases (25%);

3) Sepsis in 5 cases (25%); 

4) Severe asphyxia in 2 cases (10%); and

5) unexplained in 2 cases (16%). 

There wasn’t any significant relation between bacterial colonization of cervical sampling and neonatal death by using Chi-square test (RR=1.09, 95% CI: 0.44-2.73, p=0.840). Also, there was no relation between chorioamnionitis and neonatal death by using Chi-square test (RR=1.74, 95%CI: 0.46-6.64, p=0.42). But, there was a significant relationship between neonatal blood culture and neonatal death (RR=10.87, 95%CI: 5.89-19.71, p<0.001). Blood culture was positive in 25% of live and 0.6% of dead neonates. There wasn’t any significant relationship between positive neonatal blood culture and duration of PPROM until endocervical sampling (p=0.151). All the mothers who delivered neonates with positive blood culture had positive endocervical culture too (Table IV). 

The causes of neonatal sepsis were gram-negative bacillus in 5 cases (66.6%), gram-positive cocci in one case (16.7) and Candida in one case (16.7%).

**Table I:** The relation between chorioamnionitis and duration between PPROM up to endocervical sampling neonatal outcomes

**Chorioamnionitis**	**Duration between PPROM up to endocervical sampling**			**Total **	**p-value** *
	**>24 hr**	**12-24 hr**	**<12 hr**		
Positive	6 (28.6 )	0 (0)	6 (3.7)	12 (6.0)	<0.001
Negative	15 (71.4)	18 (100)	155 (96.3)	188 (94)	
Total	21 (100)	18 (100)	161 (100)	200 (100)	

Data presented as n (%)

PPROM: Premature rupture of membrane

* Chi-square test

**Table II:** The antibiogram for cultured microorganisms

**Result of culture**	**Total **	**No sensitivity **	**Penicillin+ cefotaxime+ cephalosporin**	**Cefotaxime+ macrolide**	**Cefotaxime+ cephalosporin**	**Cefotaxime**	**Penicillin+ cephalosporin+ macrolide**	**Cephalosporin+ macrolide**	**Penicilli+ macrolide**	**Penicillin+ cephalosporin**	**Macrolide **	**Cephalosporin **	**Penicillin **
E. coli Escherichia coli	33 (100)	9 (27.3)	0 (0)	0 (0)	6 (18.2)	7 (21.2)	1 (3)	2 (6.1)	1 (3)	1 (3)	2 (6.1)	4 (12.1)	0 (0)
Staphylococci epidermis	20 (100)	11 (55)	0 (0)	0 (0)	0 (0)	0 (0)	0 (0)	3 (15)	0 (0)	3 (15)	0 (0)	2 (10)	1 (5)
Staphylococci saprophyticus	17 (100)	8 (47.1)	1 (5.9)	0 (0)	0 (0)	0 (0)	0 (0)	0 (0)	0 (0)	4 (23.5)	0 (0)	3 (17.6)	1 (5.9)
Enterococcus	17 (100)	6 (35.3)	0 (0)	0 (0)	0 (0)	1 (5.9)	1 (5.9)	0 (0)	1 (5.9)	3 (17.6)	0 (0)	0 (0)	5 (29.4)
Enterobacter	4 (100)	1 (25)	0 (0)	1 (25)	0 (0)	0 (0)	0 (0)	0 (0)	0 (0)	0 (0)	0 (0)	2 (50)	0 (0)
Klebsiella pneumonia	12 (100)	4 (33.3)	0 (0)	0 (0)	0 (0)	5 (41.7)	0 (0)	0 (0)	0 (0)	0 (0)	0 (0)	3 (25)	0 (0)
Klebsiella oxytoca	3 (100)	1 (33.3)	0 (0)	0 (0)	0 (0)	1 (33.3)	0 (0)	0 (0)	0 (0)	0 (0)	0 (0)	1 (33.3)	0 (0)
Staphylococci areus	4 (100)	2 (50)	0 (0)	0 (0)	0 (0)	0 (0)	0 (0)	0 (0)	0 (0)	1 (25)	0 (0)	0 (0)	1 (25)
Streptococci alpha hemolytic	1 (100)	0 (0)	0 (0)	0 (0)	0 (0)	0 (0)	0 (0)	0 (0)	0 (0)	0 (0)	0 (0)	0 (0)	1 (100)
Coagulase Negative Staphylococci	1 (100)	1 (100)	0 (0)	0 (0)	0 (0)	0 (0)	0 (0)	0 (0)	0 (0)	0 (0)	0 (0)	0 (0)	0 (0)
Pseudomonas	3 (100)	0 (0)	0 (0)	0 (0)	1 (33.3)	1 (33.3)	0 (0)	0 (0)	0 (0)	0 (0)	0 (0)	1 (33.3)	0 (0)
Candida	18 (100)	18 (100)	0 (0)	0 (0)	0 (0)	0 (0)	0 (0)	0 (0)	0 (0)	0 (0)	0 (0)	0 (0)	0 (0)
Serratia	1 (100)	0 (0)	0 (0)	0 (0)	0 (0)	1 (100)	0 (0)	0 (0)	0 (0)	0 (0)	0 (0)	0 (0)	0 (0)
Acintobacter	1 (100)	1 (100)	0 (0)	0 (0)	0 (0)	0 (0)	0 (0)	0 (0)	0 (0)	0 (0)	0 (0)	0 (0)	0 (0)
Streptococci beta hemolytic	1 (100)	0 (0)	0 (0)	0 (0)	0 (0)	0 (0)	0 (0)	0 (0)	0 (0)	0 (0)	0 (0)	1 (100)	0 (0)
Total	136 (100)	62 (45.6)	1 (0.7)	1 (0.7)	7 (5.1)	16 (11.7)	2 (1.5)	5 (3.7)	2 (1.5)	12 (8.8)	2 (1.5)	17 (12.5)	9 (6.6)

All data presented as n (%).

**Table III:** Relation of NICU admission and maternal endocervical culture in PPROM cases

**Need to NICU admission**	**Maternal endocervical culture**		**Total**	**p-value** *
	**Positive culture**	**Negative culture**		
Positive	74 (54.4)	21 (32.8)	95 (47.5)	0.004
Negative	62 (45.6)	43 (67.2)	105 (52.5)	
Total	136 (100)	64 (100)	200 (100)	

All data presented as n (%)

* Fisher exact test

**Table IV:** Comparison of neonatal blood culture with maternal endocervical culture

**Cases **	**Maternal endocervical culture**	**Neonatal blood culture**	**Mean gestational age (w)**	**Neonatal outcomes**
1	Escherichia coli	Staphylococci epidermis	34	Died
2	Escherichia coli	Klebsiella pneumonia	27	Died
3	Staphylococci epidermis	Klebsiella pneumonia	33	Died
4	Staphylococci epidermis	Escherichia coli	33	Died
5	Enterococus	Klebsiella pneumonia	28	Died
6	Pseudomonas aeroginosa	Candida species	28	Alive

## Discussion

Results of this study showed that 136 cases (68%) of PPROM patients had a positive endocervical culture which mainly consists of gram-negative (31%) microorganisms and then, 29% gram-positive and 8% fungal species. The most frequent pathogens in endocervical culture were E. coli (24.2%) and then Staphylococci epidermis (14.7%), Staphylococci sapraphitices (12.5%), Enterococus (11.7%) and Candida (11.7%). just 3 cases of GBS were identified. So, the results of current study were totally different from some studies of western countries like the study of Lajos in Brazil (7) and Loeb (26) which GBS was the most frequent pathogens in the endocervical culture of PPROM women in their study. In the study which was performed by Lajos and colleagues, 212 PROM cases between 24-42 wk of gestation were evaluated and they found GBS was the most frequent microorganism in endocervical culture (7). 

In the study of Loeb and colleagues, they evaluated 300 PPROM cases between 20-25 wk of gestation (24); may be lower gestational age in their study is the cause of difference compared to the present study. Maybe there were different sampling method, different method for transferring the samples to the lab and also different culture media in these studies; which can affect the results but; in Lajos in Brazil (7) which used unspecialized culture media, again GBS was the most frequent microorganism (9.4%).

In the similar study of Dechen and colleagues which was done in India (27); the positive vaginal culture results of GBS was about 4.7% and the most common organisms were candida species (36%), staphylococc areus (8%) and enterococcus (8%), which the results of current study were completely different with them. This variation may be due to different geographic status which may had a role in bacterial colonization of genital tract. In one systematic review in 2014, the most common pathogens were staphylococcus (37.6%) and E. coli (11.9%) which was nearly similar to our results (16).

In the current study, coagulase negative Staphylococcus had a rare prevalence and it was positive just in one case (0.7%), but in the study which was done in Saudi Arabia about 24.2% of cultures was positive in terms of this micro organism but similar to current study the prevalence of klebsiela pneumonia and Enterococcus were about 12.9% and 11.3% respectively (2). In the study of Kerur and colleagues which was done in India, the results were similar to current study; E. coli and klebsiela were the most common pathogen 38.2% and 4.9% respectively and GBS was found just in one case (0.9%) (28).

In one study which performed in Kerman (another city of Iran) prevalence of GBS and anaerobic pathogens in vaginal culture of PPROM women were about 5% and 1% respectively which the prevalence of these pathogens were about twofold higher compared to the results of current study (14); these differences could be due to different bacterial genital Flora from different population even in the same country (24).

In terms of drug sensitivity of colonized bacteria in the study of Zeng and colleagues which performed a systematic review in this field, they reported that most staphylococcus species (areus and epidermis) were resistant to penicillin (66-100%), except for cloxacilin (16). In the current study, most staphylococcus species which were common bacterial colonization in endocervical culture were resistant to penicillin and macrolide (55% and 47.1%, for staphylococcus epidermis and saprophyticus, respectively); that was consistent with the findings of Zeng and coworkers. In the present study, E. coli which was one of the most frequent microorganisms in endocervical culture of PPROM cases, had no resistance to penicillin, but had resistance to cephalosporin, cefotaxime or macrolide in 40% of cases. This result was different from Zeng and co-worker study which showed more sensitivity to cephalosporins (16). This inconsistency could be explained by different drug sensitivity in various populations.

In the current study, blood culture was positive in about 6 neonates (3%) which 66.6% of these cases were gram-negative bacillus (klebsiella and E. coli) and just in one case gram-positive cocci and Candida (16.7%) were seen. In the study of Stoll and colleagues in USA; the most common pathogen of blood culture in preterm neonates (delivered from PPROM mothers) was E. coli which was consistent with the result of current study (17).

These results were different from the result of Zeng which 7.6% of neonates had positive blood culture and gram-positive pathogens were responsible for about 58.5% of these results and gram-negative bacillus existed in about 33.8% of neonatal blood cultures (16). Various common pathogens of vaginal flora and also different bacterial colonization of NICU environment in different communities and different methods of sample collection, transportation and storage could be the cause of different results. 

**Limitation**

One of the limitations of current study was that we didn’t evaluate the frequency of Chlamydia and mycoplasma in the patients, however, it was due to some important reasons; first, these microorganisms are not the normal genital flora, and second, the detection method for these two microorganisms are so complex and different and their culture media were not available for us. With regard to different bacterial genital Flora in different society even in the same country, it might be better than in PPROM cases, prophylactic antibiotics administered according to the most prevalent genital tract bacterial colonization and especially environmental pathogens of each community that the last factor (environmental pathogens) may have a cardinal role in neonatal sepsis. It is recommended that more studies should be performed to support the best choice of prophylactic antibiotics in PPROM patients according to the most prevalent bacterial colonization and neonatal blood culture.

## Conclusion

Although high prevalence of genital tract bacterial colonization was seen in the PPROM pregnant women; the GBS colonization wasn’t prevalent in this study. So, it might be better that prophylactic antibiotic administration is considered according to the most prevalent microorganism of endocervical bacterial colonization. Although vaginal bacterial colonization could increase the NICU admission, it had no effect on the prevalence of chorioamnionitis, neonatal sepsis and mortality. Important factors in neonatal mortality were positive neonatal blood culture and neonatal sepsis.

## Note

***This article extracted from M.D. Thesis. (Asieh Maleki)***
